# Sperm miRNAs— potential mediators of bull age and early embryo development

**DOI:** 10.1186/s12864-020-07206-5

**Published:** 2020-11-16

**Authors:** Chongyang Wu, Patrick Blondin, Christian Vigneault, Rémi Labrecque, Marc-André Sirard

**Affiliations:** 1 Centre de Recherche en Reproduction, Développement et Santé Intergénérationnelle (CRDSI), Département des Sciences Animales, Faculté des Sciences de l’Agriculture et de l’Alimentation, Université Laval, Québec, Québec Canada; 2 L’Alliance Boviteq, Saint-Hyacinthe, Québec, Canada

**Keywords:** Paternal age, Sperm miRNAs, Embryonic development, Dairy bull, Epigenetics

## Abstract

**Background:**

Sperm miRNAs were reported to regulate spermatogenesis and early embryonic development in some mammals including bovine. The dairy cattle breeding industry now tends to collect semen from younger bulls under high selection pressure at a time when semen quality may be suboptimal compared to adult bulls. Whether the patterns of spermatic miRNAs are affected by paternal age and/or impact early embryogenesis is not clear. Hence, we generated small non-coding RNA libraries of sperm collected from same bulls at 10, 12, and 16 months of age, using 16 months as control for differential expression and functional analysis.

**Results:**

We firstly excluded all miRNAs present in measurable quantity in oocytes according to the literature. Of the remaining miRNAs, ten sperm-borne miRNAs were significantly differentially expressed in younger bulls (four in the 10 vs 16 months contrast and six in the 12 vs 16 months contrast). Targets of miRNAs were identified and compared to the transcriptomic database of two-cell embryos, to genes related to two-cell competence, and to the transcriptomic database of blastocysts. Ingenuity pathway analysis of the targets of these miRNAs suggested potential influence on the developmental competence of two-cell embryos and on metabolism and protein synthesis in blastocysts.

**Conclusions:**

The results showed that miRNA patterns in sperm are affected by the age of the bull and may mediate the effects of paternal age on early embryonic development.

**Supplementary Information:**

The online version contains supplementary material available at 10.1186/s12864-020-07206-5.

## Background

With genomic selection technologies, dairy cattle breeding companies can now use bulls for reproductive purpose at early pubertal stages with high reliability [1]. Compared to waiting until five years of age for progeny testing, this practice dramatically increases the speed of genetic gain by reducing generation intervals.

Puberty in bulls, which is initiated by systemic information of the neuroendocrine system, is defined as the age when the first ejaculation containing over 50 × 10^6^ million spermatozoa with at least 10% progressive motility is observed [2]. It is thus predictable that semen collected from younger bulls has lower sperm count, lower motility, and results in lower IVF rates compared to sperm collected from mature bulls [3]. A lack of functional mitochondria in sperm of prepubertal bulls was also observed which may explain the reduced motility of sperm at this age [3]. It would be interesting to identify factors that could potentially be affected by paternal age in order to better understand the effects of using younger bulls on embryo and offspring development.

During fertilization, sperm transfer not only the paternal genome, but also a variety of epigenetic information, including DNA methylation, histone retention and modifications, as well as small non-coding RNAs (sncRNAs) [4]. In contrast to genetic information which is relatively stable, epigenetic factors conveyed by male gametes are more vulnerable to environmental and physiological changes and are able to affect offspring health transgenerationally [5, 6]. Increasing evidence suggests that DNA methylation patterns, miRNAs, and modifications of retained histones are related to bull fertility status [7–10]. Moreover, differentially methylated regions (DMRs) were observed in sperm of bulls at different pubertal stages [3, 11, 12]. However, following DNA methylation reprogramming during embryogenesis, only a few DMRs were retained [11], suggesting that other epigenetic factors, such as sncRNAs, may potentially function as paternal transgenerational inheritance factors.

Sperm sncRNAs were initially regarded as residues of spermatogenesis. However, recent studies discovered that sncRNAs, such as miRNAs and tsRNAs, are sensitive to environmental, metabolic, and psychological stresses [6, 13, 14]. Such programming could then transgenerationally impact preimplantation embryos [15] and even offspring development [16]. Sperm cells carrying different miRNA and/or endo-siRNA deficiencies in *Dicer* and *Drosha* conditional knockout mice could still fertilize oocytes but the developmental potential of the resulting embryos was significantly reduced [17]. Individual miRNA studies also concluded that sperm-borne miR-34c is required for the first cell division in mouse embryos via targeting of the Bcl-2 mRNA [18]. In bovine spermatozoa, the microRNA pattern was demonstrated by deep sequencing [19]. However, whether the expression pattern of sperm-borne sncRNAs varies during the sexual maturation of young bulls is not clear and their functions in early embryonic development have not been described yet.

The objective of this study was to assess the potential differences between small non-coding RNAs in semen of bulls collected at 10, 12, and 16 months of age (pre-, peri-, and post-pubertal stages, respectively). More than a thousand bovine miRNAs were identified, and ten sperm-borne miRNAs were significantly differentially expressed in younger bulls. These miRNA targets are associated with developmental and metabolic pathways such as TGF-β, PI3K/AKT, oxidative phosphorylation and mitochondrial dysfunction in pre-implantation embryos, thus may mediate paternal age effects on early embryonic development.

## Results

### Bovine sperm small RNA purification

Semen was collected from the same four bulls at the ages of 10, 12, and 16 months, representing the pre-, peri- and post-pubertal stages, respectively. Semen quality was analyzed, bulls of 10 months old ejaculated significantly less sperm (*p*-value < 0.01) and had a tendency of lower concentration (*p*-value = 0.19) and lower post-thaw progressive motility rate compared to 12 and 16 months old (*p*-value = 0.21), while no difference was observed in total motility among three ages (Supplementary Material, Fig. S1).

**Fig. 1:**
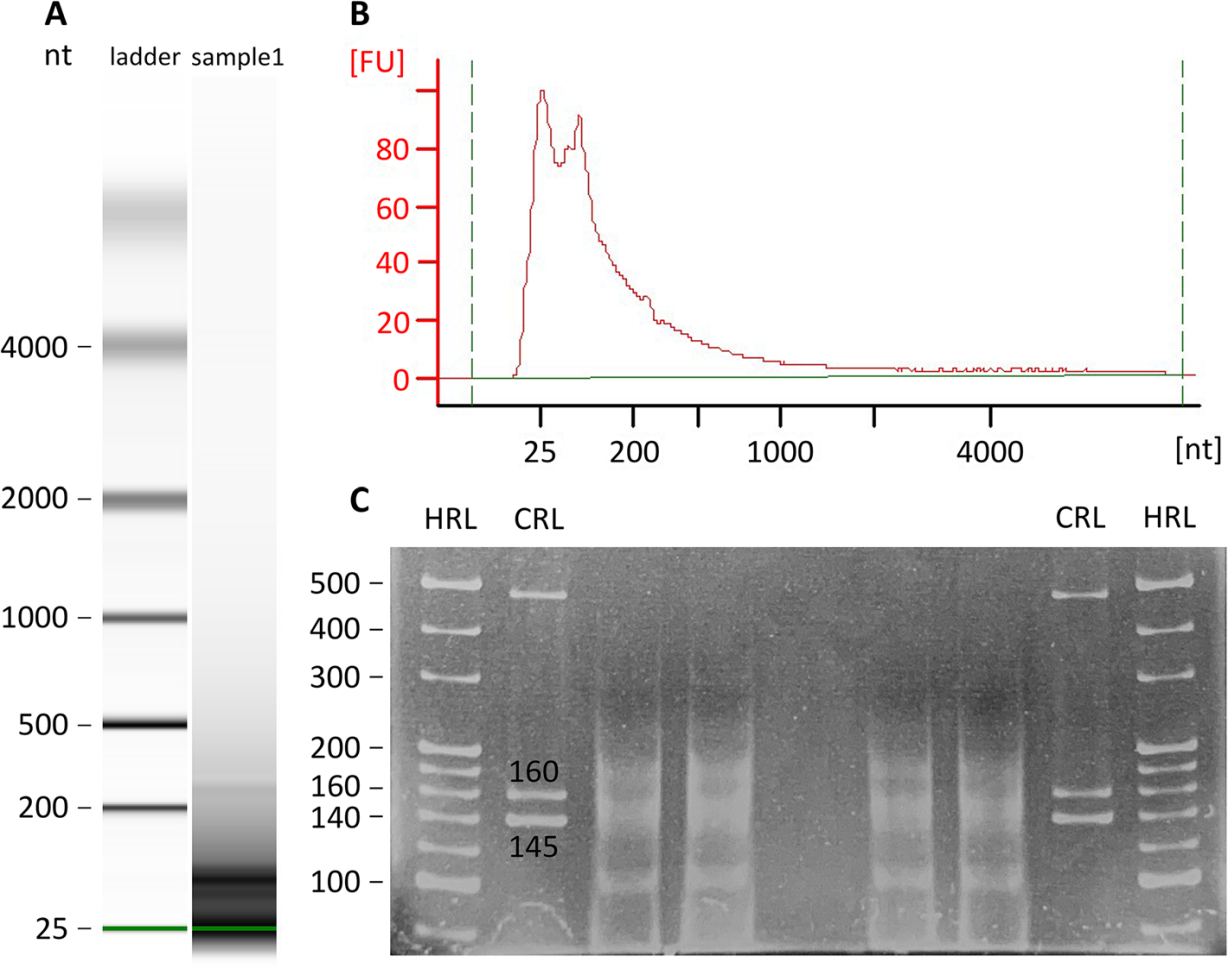
RNA patterns of sperm from bull #06622 at 10 months old. **a, b:** Bioanalyzer results of total RNA extracted from sperm. **c:** Small RNA libraries with adapters of 125 nt (small noncoding RNAs as well as miRNAs were cut and purified for sequencing). HRL: High Resolution Ladder. CRL: Custom RNA Ladder. Summary of all RNA patterns of sperm was shown in Supplementary Material (Fig. S2)

An average of 87 ng total RNA with RIN of 2.45 ± 0.015 was extracted from five straws for each age group (Supplementary Material, Table S1). Bovine spermatozoa from semen collected at different pubertal stages displayed similar overall RNA profiles. The RNA profile of bull #06622 at 10 months old is shown as an example: most fragments were shorter than 200 nt illustrating the sperm specific short-length RNA pattern (Fig. 1a and b). During library construction, sperm total RNA was ligated to adapters 125 nt long and reverse transcribed. Following amplification, the cDNA libraries were purified by TBE gel, and fragments between 145 and 400 nt, which represented small non-coding RNAs, were cut for sequencing (Fig. 1c).

### Small non-coding RNA patterns in spermatozoa from bulls at different pubertal stages

**Fig. 2:**
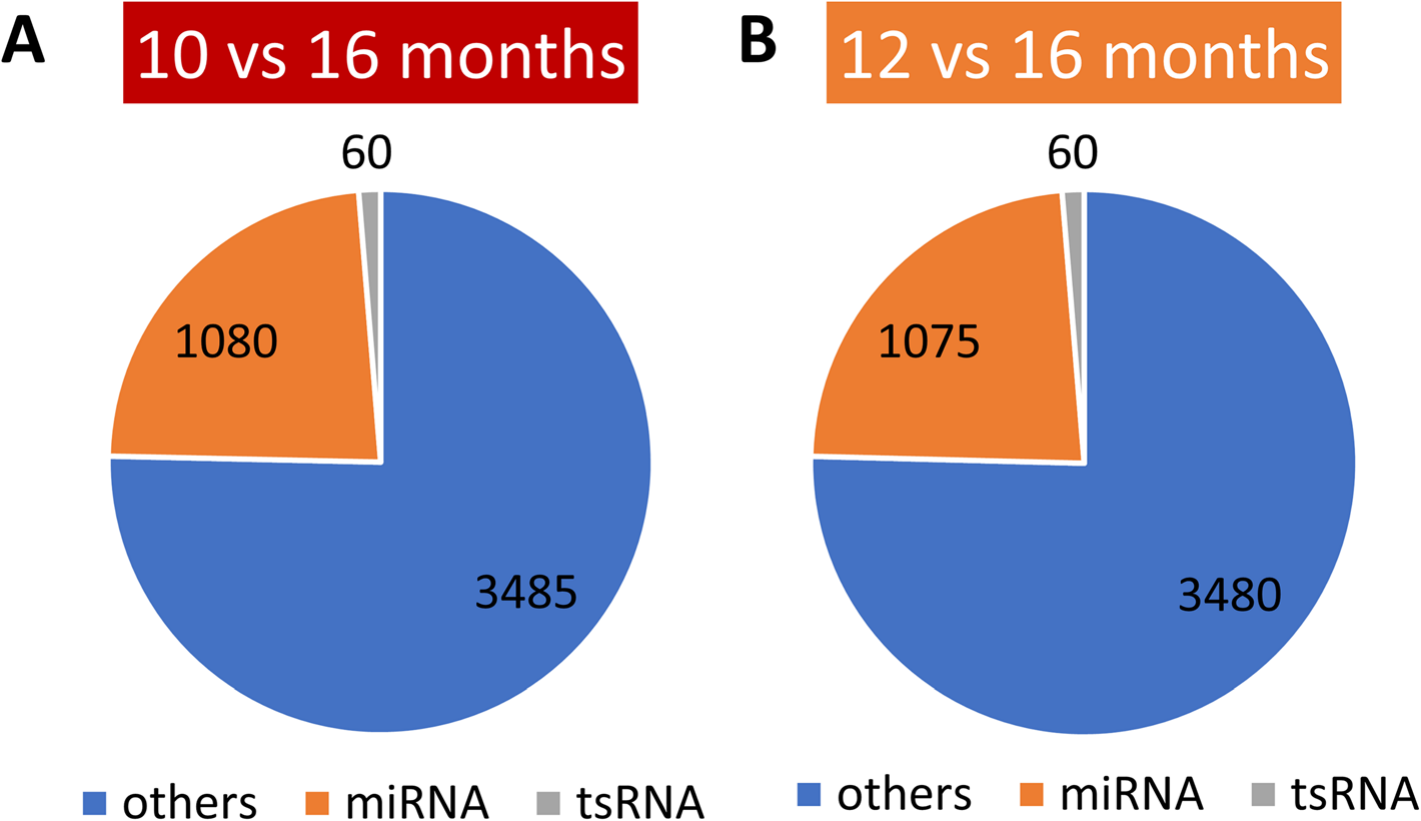
Pie charts of RNA distribution in sperm. **a**: The distribution of RNAs that are expressed in sperm from bulls at 10 vs 16 months. **b**: The distribution of RNAs that are expressed in sperm from bulls at 12 vs 16 months

A total of 4625 and 4615 unique sequences were identified in the 10 vs 16 months and the 12 vs 16 months contrasts, respectively, including 1080 miRNAs and 60 tsRNAs for the former as well as 1075 miRNAs and 60 tsRNAs for the latter group, which were mapped to the bovine database (Fig. 2a and b). Differential expression analysis for the four bulls compared to themselves at a different age resulted in two miRNAs significantly overexpressed and four miRNAs underexpressed in spermatozoa of bulls at 10 months compared to 16 months of age. Similarly, three miRNAs and one tsRNA were upregulated while five miRNAs were down regulated at 12 months compared to 16 months of age. After filtering out the small non-coding RNAs known to be present in maternal gametes [20, 21], ten differentially expressed miRNAs were selected from the two contrasts for further functional analysis (Table 1, and Supplementary Material, Table S2).

**Table 1:** Differentially expressed sperm-borne miRNAs in two contrasts

10 vs 16 months			12 vs 16 months		
Candidate miRNA	Fold Change	***p***-value	Candidate miRNA	Fold Change	***p***-value
bta-mir-19b	2.20	0.0029	bta-mir-759	4.99	0.0008
bta-mir-133a	1.89	0.0354	bta-mir-2284z	4.44	0.0213
bta-mir-2285b	−3.45	0.0273	bta-mir-34b	2.14	0.0227
bta-mir-2405	−1.52	0.0436	bta-miR-2334	−4.35	0.0230
			bta-mir-2285e	−2.44	0.0105
			bta-mir-302a	−2.38	0.0401

### Paternal age effects on two-cell embryos

The predicted target genes of these miRNAs were obtained from TargetScan (release 7.2) [22] to explore the potential paternal age effects on early embryo development. Ingenuity pathway analysis (IPA) of target genes expressed in two-cell embryos according to our previous study [23] indicated that the top five canonical pathways affected by paternal age were mainly related to metabolic and developmental pathways (Table 2). For example, TGF-β signaling and Rho family GTPase signaling were activated in the 10 vs 16 months group, while PI3K/AKT signaling, Insulin receptor signaling, and AMPK signaling were activated and PTEN signaling was inhibited in the 12 vs 16 months group. Insulin-like growth factor − 1 signaling activation was included in the top five list for each contrast. Thus, miRNAs from sperm of younger bulls may potentially impact the metabolism and development of two-cell embryos.

**Table 2:** Top five canonical pathways assembled from targets of differentially expressed sperm-borne miRNAs in two-cell embryos

10 VS 16 months			12 VS 16 months		
Canonical Pathways	-log(*p*-value)	z-score	Canonical Pathways	-log(*p*-value)	z-score
TGF-β Signaling	11.4	4.95	PI3K/AKT Signaling	10.1	2.402
Signaling by Rho Family GTPases	10.8	6.565	PTEN Signaling	7.39	−3.43
Huntington’s Disease Signaling	10.1	2.333	Insulin Receptor Signaling	6.67	2
IGF-1 Signaling	9.59	4.226	IGF-1 Signaling	6.31	3.266
Cardiac Hypertrophy Signaling	9.13	5.515	AMPK Signaling	6.21	2.596

Note: A Z-score ≥ 2 means predicted activation, while a z-score ≤ −2 predicts inhibition

### Paternal age effects on developmental competence of two-cell embryos

Early embryonic cleavage kinetics is one of the most important indicators of developmental competence with embryos cleaving sooner following fertilization possessing higher developmental capacity [24, 25]. A data set was previously obtained by comparing the mRNA content of two-cell embryos that had divided shortly after fertilization (competent) to later dividing embryos (less competent). Transcripts present at the two-cell stage are either translated rapidly between the two- and eight-cell stages, or simply degraded by the eight-cell stage, therefore creating a two to three-day window for being targeted by sperm-borne or other miRNAs [26–28]. In our previous study, we identified 1368 gene transcripts that were differentially expressed in fast- vs slow-cleaving two-cell embryos [23]. In the present study, 424 and 332 of these 2-cell developmental competence related genes were targets of differentially expressed miRNAs in sperm from bulls at 10 vs 16 months, and 12 vs 16 months of age, respectively. Furthermore, IPA revealed that the top five canonical pathways of candidate genes targeted by differentially expressed miRNAs in the 10 vs 16 months group were related to embryonic stem cell pluripotency, biosynthetic process, and thrombin signaling (Table 3). Meanwhile, in the 12 vs 16 months group, the top five canonical pathways activated by the differentially expressed miRNAs were PI3K signaling, iCOS-iCOSL signaling, inhibition of angiogenesis by TSP1, non-small cell lung cancer signaling, and telomerase signaling (Table 3). In this case, spermatozoa ejaculated from younger bulls could influence the metabolism, pluripotency, and immune response related pathways to impact the developmental capacity of the two-cell embryos immediately or between the two- and eight-cell stages.

**Table 3:** Top five canonical pathways assembled from targets of differentially expressed sperm-borne miRNAs correlated with two-cell embryo competence

10 VS 16 months			12 VS 16 months		
Canonical Pathways	-log(*p*-value)	z-score	Canonical Pathways	-log(*p*-value)	z-score
Mouse Embryonic Stem Cell Pluripotency	4.81	2.111	PI3K Signaling in B Lymphocytes	3.62	3
Superpathway of Inositol Phosphate Compounds	3.63	3.207	iCOS-iCOSL Signaling in T Helper Cells	3.22	2.646
Role of NANOG in Mammalian Embryonic Stem Cell Pluripotency	3.61	2.646	Inhibition of Angiogenesis by TSP1	2.79	2
Thrombin Signaling	3.41	2.714	Non-Small Cell Lung Cancer Signaling	2.68	2.236
3-phosphoinositide Biosynthesis	3.21	2.887	Telomerase Signaling	2.61	2.449

Note: A Z-score ≥ 2 means predicted activation, while a z-score ≤ −2 predicts inhibition

### Paternal age effects on blastocysts

Due to the relative short half-life of miRNAs, a direct effect of sperm-borne miRNAs at the blastocyst stage is questionable. However, it is possible that the miRNA targets will transmit the paternal age effects to later stages or create a context that will have an impact a few cell cycles later at the blastocyst stage. To explore these possibilities, we used the transcriptomic data of blastocysts produced in vitro from sperm of younger bulls [29]. In that study, we used IPA to predict the upstream regulators of the differentially expressed genes (DEGs) in blastocysts from younger bulls in a very similar age design as the present study. Among the upstream regulators previously identified, three were targets of miRNAs that were differentially expressed at 10 vs 16 months, and four were targets of miRNAs differentially expressed at 12 vs 16 months (Table 4). Interestingly, two of the upstream regulators (RICTOR and KDM5A) were common to the two groups. IPA of the downstream targets of these common regulators demonstrated that four of the top five canonical pathways were common to the two contrasts, e.g. oxidative phosphorylation, mitochondrial dysfunction, EIF2 signaling, and sirtuin signaling pathway (Table 5). These pathways were all closely related to the metabolism of blastocysts.

**Table 4:** Upstream regulators of the blastocyst transcriptome that are targets of sperm borne miRNAs

Upstream regulators in 10 vs 16 months	Downstream targets	Upstream regulators in 12 vs 16 months	Downstream targets
RICTOR	ATP5MF, ATP6V0A4, ATP6V1D, etc. 24 genes	RICTOR	ATP5PD, ATP6V0D2, COX7A2L, etc. 18 genes
KDM5A	ACTC1, ATP6V1D, ATP6V1F, etc. 15 genes	STK11	ATIC, AVIL, CCNA2, etc. 14 genes
CREB1	CHRNA1, CNN1, CSRP2, etc. 21 genes	KDM5A	COQ7, MCM3, MYL4, etc. 7 genes
		AREG	CCNA2, CENPF, PRC1, etc. 5 genes

**Table 5:** Top five canonical pathways potentially affected by bull age-related sperm-borne miRNAs in blastocysts

10 VS 16 months		12 VS 16 months	
Canonical Pathways	-log(*p*-value)	Canonical Pathways	-log(*p*-value)
Oxidative Phosphorylation	17.3	Oxidative Phosphorylation	12.9
Mitochondrial Dysfunction	16.6	Mitochondrial Dysfunction	11.1
EIF2 Signaling	7.54	Sirtuin Signaling Pathway	6.32
Sirtuin Signaling Pathway	5.58	EIF2 Signaling	4.39
TCA Cycle II (Eukaryotic)	4.75	Pyrimidine Deoxyribonucleotides De Novo Biosynthesis I	3.17

## Discussion

### Function of sperm miRNA in early embryo development

**Fig. 3:**
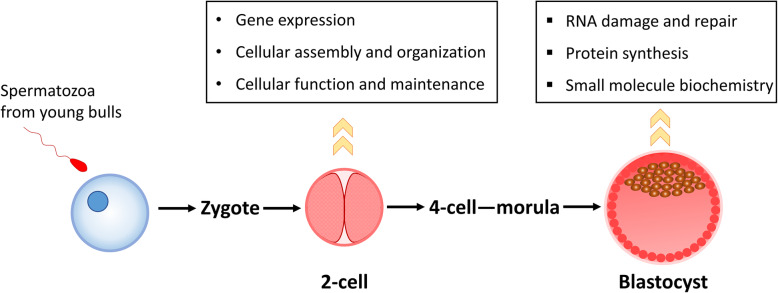
Molecular and cellular functions affected by paternal age in embryos. Listed functions at 2-cell stage are overlapped top five molecular and cellular functions from IPA analysis among the two contrasts (10 vs 16 months and 12 vs 16 months) of the two databases (transcriptomic data of 2-cell embryos and genes related to 2-cell embryo competence). Listed functions at blastocyst stage are overlapped top five molecular and cellular functions from IPA analysis between the two contrasts (10 vs 16 months and 12 vs 16 months) of the blastocyst database

The function of sperm-borne miRNAs was ignored for a long time as they were thought to simply be remnants or byproducts of spermatogenesis. Recently, promising results were obtained regarding the regulation of sperm-borne miRNAs in early embryonic development [17, 18, 30]. One of the six miRNAs (miR-34c) expressed in sperm and zygotes, but not in oocytes and preimplantation embryos, was required for the first cell division in vitro [18]. However, in vivo research using miR-34b/c and miR-449 double knockout (miR-dKO) mice demonstrated that the two miRNA clusters were indispensable for spermatogenesis and fertility, but not for fertilization or preimplantation development [30]. The conflicting results were mainly due to the off-target effects of the inhibitor of miR-34c used in the former study, indicating that other sperm-borne miRNAs might be involved in early embryonic development. This assumption was further verified by germline-specific Dicer and Drosha conditional knockout in mice, which resulted in failure of zygotic genome activation and embryonic development [17]. One member of this miRNA family, miR-34b, was differentially expressed in the sperm of young bulls; however, the blastocyst rates were not significantly affected [29], indicating that this miRNA does not have a lethal influence on bovine embryos. Molecular and cellular function analysis using IPA predicts that the differentially expressed sperm-borne miRNAs from young bulls will potentially influence the gene expression, cellular assembly and organization, and cellular function and maintenance at 2-cell stage, while later at blastocysts stage, these miRNAs will be likely to affect RNA damage and repair, protein synthesis, and small molecule biochemistry (Fig. 3). As in other species, it is therefore quite possible that the dominant biofunctions of these miRNAs is to carry paternal epigenetic effects.

### sncRNAs as epigenetic inheritance factors

Epigenetic factors, including DNA methylation, non-coding RNAs, histone modification, and others, are more sensitive to environmental conditions compared to genetic information. Epigenetic information in sperm was proven to translate the environmental experiences of the father who could then transfer these acquired traits to his progeny [31]. In a previous study from our lab, sperm from prepubertal bulls were characterized to bear a different DNA methylation pattern compared to sperm from post-pubertal bulls [11]. Moreover, in the mouse, the global reprogramming during early embryonic development does not erase all methylation marks from the sperm, leaving a few regions for inter or transgenerational inheritance [32]. In our study mentioned above, there were over 2600 differentially methylated regions between sperm of bulls at 10 and 16 months of age, including one of them located on an imprinted gene [11]. Recently, contradictory evidence cast some doubts on whether sperm DNA methylation is either the primary or the persistent epigenetic carrier of paternally acquired traits [33, 34]. Hence, other epigenetic factors such as sperm-borne sncRNAs are gaining more and more attention as transgenerational inheritance factors from the paternal side. The direct causal relationship between sperm RNAs and paternally inherited characteristics was first reported in a study in which total sperm RNA from traumatized mice was injected into wild type zygotes resulting in offspring with altered behavior and metabolism [14]. Catenin β1 (Ctnnb1), which is involved in stress pathways, was validated as a target of miR-375 which was differentially expressed in mentally stressed mice. The expression of Ctnnb1 was inhibited in F2 offspring [14]. This finding was further supported by various independent studies on the transgenerational inherited paternal acquired traits following mental stress [35], toxicant exposure [6], and diet changes [16]. Moreover, paternal age is another factor related to the health of offspring. Advanced paternal age in human increases the probability of genetic disorders such as schizophrenia, bipolar disorder, and autism, as reviewed by Sharma et al. [36]. Given that increased age generally also includes exposure to other complex environmental factors, such as physiological age, nutrition and lifestyle, genetics stability, hormones, senile diseases, oxidative stress, and telomere shortening [36], paternal effects of advanced age depend on both genetic and epigenetic changes. However, in our study of a relatively short age span in bulls (from 10 to 16 months old), epigenetic changes, especially sncRNAs, seemed to be affected by a young paternal age. In this study, fourteen miRNAs and one tsRNA were differentially expressed in sperm of younger bulls. Considering the vast amount of miRNAs stored in oocytes, sperm miRNAs that are also present in oocytes may have limited purposes, if any, in fertilization or early embryogenesis [37]. Thus, we finally selected ten miRNAs that were exclusively expressed in paternal gametes, based on published transcriptomic data of bovine oocytes [20, 21], to focus on specific sperm effects.

Several of the paternal differentially expressed sncRNAs in our study were associated with environmentally induced epigenetic inheritance of metabolic traits. A high-fat and/or high-sugar diet (Western-like diet) induced obesity and type II diabetes in mice (F0) and these traits were inherited by the resulting progenies [38]. The expression of several miRNAs, including miR-19b was inhibited in sperm of F0 mice [38]. Intriguingly, when miR-19b was injected into wild type one-cell embryos, the offspring (here referred as R1) exhibited similar metabolic disorders as the F0 and F1 mice, and this pathological phenotype was further inherited after mating with healthy partners [38]. In another study focusing on mental stress, offspring of running males had suppressed reinstatement of juvenile fear memory; meanwhile, the expression of sperm-borne miR-19b and miR-133a was altered in these fathers, indicating the potential involvement of these miRNAs in the effects of paternal exercise on the anxiolytic behavioral phenotype of offspring [39].

### Metabolism related pathways in early embryo development

Normal development of the early embryo promotes further later normal development. For example, in bovine embryos, a faster cleavage of the zygote into a 2-cell embryo is positively correlated to a higher probability of the embryo becoming a viable blastocyst. In the present study, the transcriptome of bovine 2-cell embryos and genes related to developmental capacity were used to assess the value of target mRNA by sperm miRNA [23]. We then used a functional analysis (IPA) of the targets of paternal age-related sperm miRNAs to find pathways that might be affected. Although IPA were not specific to cattle, but the enrichment analysis results were similar to DAVID analysis (data not shown). Moreover, IPA provides an activation z-score which infers to the activation status of biological function (activated or inhibited) [40]. Our results indicated that the pathways targeted were related to metabolism and totipotency which are immediately important for embryo quality and survival.

In the two contrasts, insulin-like growth factor 1 (IGF1) signaling was predicted to be activated by the interaction of sperm miRNA and maternal targets in 2-cell embryos generated from sperm from younger bulls (10 and 12 months), compared to 16-month-old bulls. Insulin-like growth factor 1 levels are higher in younger growing animals and possibly involved in metabolic programming [41–43]. The addition of physiological concentrations of IGF1 (10–100 ng/mL) improved the developmental competence of bovine early embryos in vitro [44–46]. In mice, a study using a high IGF1 concentration, i.e. 130 nM (201 ng/mL), which mimics the pathological condition of polycystic ovary syndrome, induced apoptosis in blastocysts via down regulation of the IGF1 receptor (IGF1R) [47] and a p53 dependant mechanism [48]. Similar results were also observed in bovine blastocysts supplemented with a supraphysiological concentration of IGF1 (1000 ng/mL), including induced apoptosis and decreased TP53 protein expression, but without inhibition of IGF1R [49]. Hence, the beneficial or adverse effects of IGF1 are concentration dependant and abnormally over activated IGF1 signaling might impair early embryogenesis.

Activation of TGF-β signaling was predicted in sperm from 10-month-old vs 16-month-old bulls. This pathway is involved in the regulation of several aspects of early embryonic development, such as embryonic patterning, cell fate determination, and dynamic movements [50]. The regulatory role of this pathway is exerted beyond the 2-cell stage in mouse embryos [51], indicating that it could possibly be programmed at the 2–8 cell stage in bovine embryos and impact blastocyst differentiation. The Rho family GTPases, a family of small signaling G proteins, are involved in sperm induced egg activation [52]. In zygotes, inhibition of cell division cycle 42 (Cdc42), a member of the Rho family, significantly decreased in vitro development to the morula or blastocyst stage [53]. The involvement of Rho family GTPases in polarisation of early mouse blastomeres was also demonstrated [54].

The IPA predicted that several insulin-related pathways were impacted by paternal age. Insulin activates its receptor to phosphorylate and recruit many substrates, including IRS and Shc, to modulate various canonical pathways, such as the PI3K/Akt, AMPK, and Ras/MAPK pathways [55, 56]. These pathways transduce the diverse effects of insulin on energy-associated cellular functions, such as glucose and lipid metabolism, protein synthesis, growth, and survival [57]. Although insulin transcripts are not detectable at the preimplantation stage in bovine embryos, the mRNA encoding insulin receptors, IGFI, and IGFII can be detected starting at the 1-cell stage [58], indicating that bovine embryos are sensitive to insulin. Similar to other mammals, cattle fed a restricted diet exhibited a reduced basal insulin concentration and improved pancreatic insulin sensitivity [59]. Hence, the insulin-related pathways predicted as activated in response to the different miRNAs in sperm from younger bulls might be associated with the prepubertal condition when energy is primarily devoted to growth, somewhat mimicking a feed restriction context. Transgenerational inheritance of paternal metabolic disorders was observed in various domestic farm animals [60]. As a consequence, embryos from younger bulls might have altered metabolism compared to embryos from adult bulls.

### Pre-implantation embryo developmental competence

Transcriptome analysis of two-cell embryos with different developmental dynamics revealed that DEGs in the earlier or later cleaving embryos were related to the cell cycle, gene expression, RNA processing, and protein degradation functions [23]. Intriguingly, some of these DEGs were targets of the miRNAs related to bull age identified in the present study. The canonical pathway analysis of this dataset demonstrated that the IP3 pathway, PI3K pathway, and pluripotency related pathways could be impacted by paternal age and could therefore affect the developmental competence of two-cell embryos. The IP3 pathway has been identified as a common mechanism to activate the metabolism of unfertilized oocytes by inducing Ca^2+^ oscillations in various species, from marine invertebrates to mammals [61]. During bovine oocyte fertilization, the IP3 pathway is required for Ca^2+^ release, and its inhibition impairs the first embryonic interphase [62]. The PI3K pathway is widely studied as a mediator of cell growth, proliferation, differentiation, and survival signals. In mouse preimplantation embryos, the p85 and p110 subunits of PI3K were expressed from the one cell to the blastocyst stage [63]. Moreover, deletion of 3-phosphoinositide-dependent protein kinase 1 (PDK1) arrested mouse embryos at the two-cell stage as a result of the inhibition of embryonic genome activation (EGA) [64]. Pluripotency is the capacity of embryonic stem cells to differentiate into all cell types of a body and is controlled by several key regulators (OCT4, NANOG, SOX2, etc.). In bovine embryos, Oct4 and Sox2 transcripts were present at the pre-EGA phase [65, 66]. Meanwhile, during the maternal-to-embryonic transition, transcripts of genes involved in the maintenance of pluripotency of embryos were also observed [67]. Hence, the paternal age-related miRNAs may potentially target pathways associated with two-cell competence to further impact early embryonic development.

In a previous study, we generated blastocysts in vitro with semen from different bulls of the same age as the ones used in the present study [29]. The transcriptome of these blastocysts was influenced by paternal age, and canonical pathways related to the DEGs resulted in altered energy production and protein synthesis. Due to their relatively short half life, miRNAs from sperm might not survive to the blastocyst stage. However, the predicted upstream regulators of DEGs identified in blastocysts could mediate their effects on the transcriptome of blastocysts. In this case, we found four and three upstream regulators that were the targets of differentially expressed miRNAs in the 10 vs 16 months and 12 vs 16 months contrasts. Intriguingly, when we identified the downstream targets of these regulators and redid the IPA analysis, the results of the small fraction were highly consistent with the overall DEGs of the blastocyst transcriptome associated with the age effect, indicating that sperm miRNAs could be directly involved in the intergenerational inheritance of factors associated with paternal age.

## Conclusion

In summary, the analysis of sncRNA in sperm from bulls at different pubertal stages revealed that the abundance of specific miRNAs could be affected by paternal age and could potentially influence the metabolism and developmental capacity of pre-implantation embryos. This perspective calls for attention on the potential consequences of using gametes from younger animals; and the miRNAs identified in this study could potentially be used to assess if a bull is mature enough to be used commercially.

## Methods

### Chemicals

All reagents used in this study were of tissue culture grade and obtained from Sigma-Aldrich (Oakville, Ontario, Canada), unless otherwise specified.

### Semen collection

All samples were obtained from The Semex Alliance (Guelph, ON, Canada), a commercial provider of bovine gametes. Semen was collected from each Holstein bull (*n* = 4, bull #03981, bull #06622, and bull #10051, bull #10085) at the ages of 10, 12, and 16 months using an artificial vagina, in compliance with the guidelines of the Canadian Council on Animal Care. Semen characteristics were examined and recorded and straws containing about 1.5 × 10^7^ spermatozoa were frozen in liquid nitrogen. Each animal served as its own control and two contrasts (10 vs 16 months and 12 vs 16 months) were created for the RNA-seq analysis described below.

### Sperm small RNA extraction

For each treatment, five straws of semen were thawed in 37 °C water for 1 min and the content was combined. After washing twice with PBS, the pellets were resuspended 1 mL Somatic lysis buffer (0.5% Triton X-100, PBS) and incubated on ice for 30 mins. After another wash with PBS, the pellets were resuspended in 60 μL PBS and each sperm suspension was added to 33.3 μL sperm lysis buffer (4 M Guanidine Thiocyanate, 5% Tween 20, 5% Triton X-100, 120 mM EDTA, and 120 mM Tris-HCl pH 8.0), 3.3 μL Proteinase K (20 mg/mL), and 3.3 μL 0.1 M DTT, then incubated for 15 min at 60 °C with gentle shaking (20 rpm). Small RNAs were extracted with the miRNeasy Mini Kit (QIAGEN, Toronto, ON, Canada, Cat. # 217004) following the manufacturer’s instructions. Briefly, 700 μL QIAzol lysis reagent were added to the previous mixture and then moved to a QIAshredder homogenizer column (QIAGEN, Toronto, ON, Canada, Cat. # 79654). After centrifugation at 13,300 rpm for 2 min at RT, the flow through was transferred to a new tube and kept at RT for 5 min to further dissociate nucleoprotein complexes. Chloroform (140 μL) was added and the samples were shaken vigorously for 15 s. The samples were left at RT for 2–3 min and then centrifuged at 12,000 g for 15 min at 4 °C. The upper aqueous phase of each sample was transferred to a new collection tube, 1.5 volume of absolute ethanol was added, and the solution was moved to an RNeasy Mini spin column to absorb the RNAs. The flow through was discarded after centrifugation and 40 μL DNase buffer (27 Kunitz units) were added directly to the middle of the membrane in the column and then incubated at RT for 15 min. The column was further washed with the provided buffer RWT once and buffer RPE twice. The column was placed into a new 2 mL collection tube and the RNAs were dissolved in 30 μL elution buffer. The integrity and concentration of sperm RNA were evaluated with an Agilent 2100 Bioanalyzer (Agilent Technologies, Palo Alto, CA).

### Small RNA library construction and solexa sequencing

The libraries of sperm small RNAs were constructed according to the manual of TruSeq Small RNA Library Prep Kit (Illumina, U.S.A., v02) with minor modifications. In brief, RNA 3′ and 5′ adapters were ligated to an average of 87 ng of sperm RNA in 5 μl nuclease-free water. The adapter-ligated RNA was then reverse transcribed in the presence of RNA RT Primer, first strand buffer, dNTP Mix, DTT, RNase inhibitor, and SupserScript II Reverse Transcriptase according to the protocol and incubated at 50 °C for 1 h. For each sample, two primers: the same RNA PCR Primer 1 and a specific RNA PCR Primer index were used for 11 cycles of amplification. The amplified RNA libraries were resolved on a 6% Novex TBE gel for 35 min at 145 V, and DNA fragments ranging from 145 to 400 bp, which represented original RNA sizes of 20 to275 bp, were cut and purified by ethanol precipitation. The pellets were dissolved in 10 μL elution buffer (10 mM Tris-HCl, pH 8.5) and the purity and concentration of the libraries were checked by NanoDrop. Sequencing was done on an Illumina Hiseq 2500 at McGill University (Montreal, QC, Canada) following the recommended protocol.

### Bioinformatics analysis of small RNAs

Sequencing data were processed for differential expression and functional analysis. Reads were quality-trimmed using Trimmomatic [68] before being aligned to a synthetic reference of all bovine miRNAs, tRNAs, and ncRNAs using bowtie. Differential expression was assessed using the DESeq [69] package. Differentially expressed miRNAs (≥1.5-fold change, *p*-value≤0.05) that were reported to be expressed at high intensity in maternal gametes [20, 21], which will dilute the original fold change of miRNAs in sperm under 1.2, were excluded. We therefore assumed that the remaining miRNAs were sperm-borne. Targets of these differentially expressed spermatic miRNAs were identified with TargetScan (release 7.2) [22]. Targets that are presented in both the of up- and down-regulated miRNAs were filtered out. The list of remaining targets was then overlapped with the transcriptomic databases from 2-cell embryos [23] and blastocysts [29]. For the functional prediction of sperm miRNAs in 2-cell embryos, databases of targets expressed at the 2-cell stage and related to 2-cell competence were created for IPA (Supplementary Materials, Tables S3, S4, S5, S6). For the functional analysis in blastocysts, upstream regulators that were predicted from the transcriptome data of blastocysts and were also targets of sperm miRNAs were selected. Downstream targets of these upstream regulators were extracted to create a new database for IPA analysis (Supplementary Materials, Tables S7 and S8).

### Statistical analyses

One-way ANOVA with Turkey posttest analyses was performed on sperm count, concentration, total motility, and post-thaw progressive motility using GraphPad Prism 6 (GraphPad Software, San Diego, CA, USA). Differences were considered statistically significant with a *p*-value < 0.05.

## Supplementary Information

**Additional file 1: Figure S1.** Semen quality of bulls at age of 10, 12 and 16 months. A: Sperm count; B: Sperm concentration; C: Total motility; D: Post-thaw progressive motility. Bars show the mean ± standard error; different letters represent *p*-value < 0.05.

**Additional file 2: Figure S2.** RNA patterns of sperm from four bulls at 10, 12, and 16-months old. A: Summary of bioanalyzer results of total RNA extracted from sperm. B: Summary of 6% Novex TBE gel results after libraries preparation. Adapters of 125 nt were added, small noncoding RNAs as well as miRNAs were cut and purified for sequencing.

**Additional file 3: Table S1.** Total RNA extracted from bovine spermatozoa. **Table S2.** Differentially expressed miRNA in the two contrasts. **Table S3.** Dataset of targets expressed in 2-cell embryo of 10vs16 months contrast for IPA. **Table S4.** Dataset of targets expressed in 2-cell embryo of 12vs16 months contrast for IPA. **Table S5.** Dataset of targets related to 2-cell embryo competence of 10vs16 months contrast for IPA. **Table S6.** Dataset of targets related to 2-cell embryo competence of 12vs16 months contrast for IPA. **Table S7.** Dataset of targets expressed in blastocyst of 10vs16 months contrast for IPA. **Table S8.** Dataset of targets expressed in blastocyst of 12vs16 months contrast for IPA.

## Data Availability

RNA-seq data were deposited in NCBI’s Gene Expression Omnibus (GEO) and are accessible through GEO series accession number: GSE147742 (https://www.ncbi.nlm.nih.gov/geo/query/acc.cgi?acc=GSE147742).

## Abbreviations

DEGs: Differentially expressed genes
DMRs: Differentially methylated regions
IGF1: Insulin like growth factor 1
IGF1R: Insulin like growth factor 1 receptor
IPA: Ingenuity pathway analysis
miRNA: microRNA
sncRNAs: Small non-coding RNAs
tsRNA: Transfer RNA-derived small RNAs
