# Supplementary figures and images for: Sperm miRNAs— potential mediators of bull age and early embryo development
Source: BMC Genomics. 2020 Nov 16;21:798. doi: 10.1186/s12864-020-07206-5 (PMC7667858; doi:10.1186/s12864-020-07206-5)

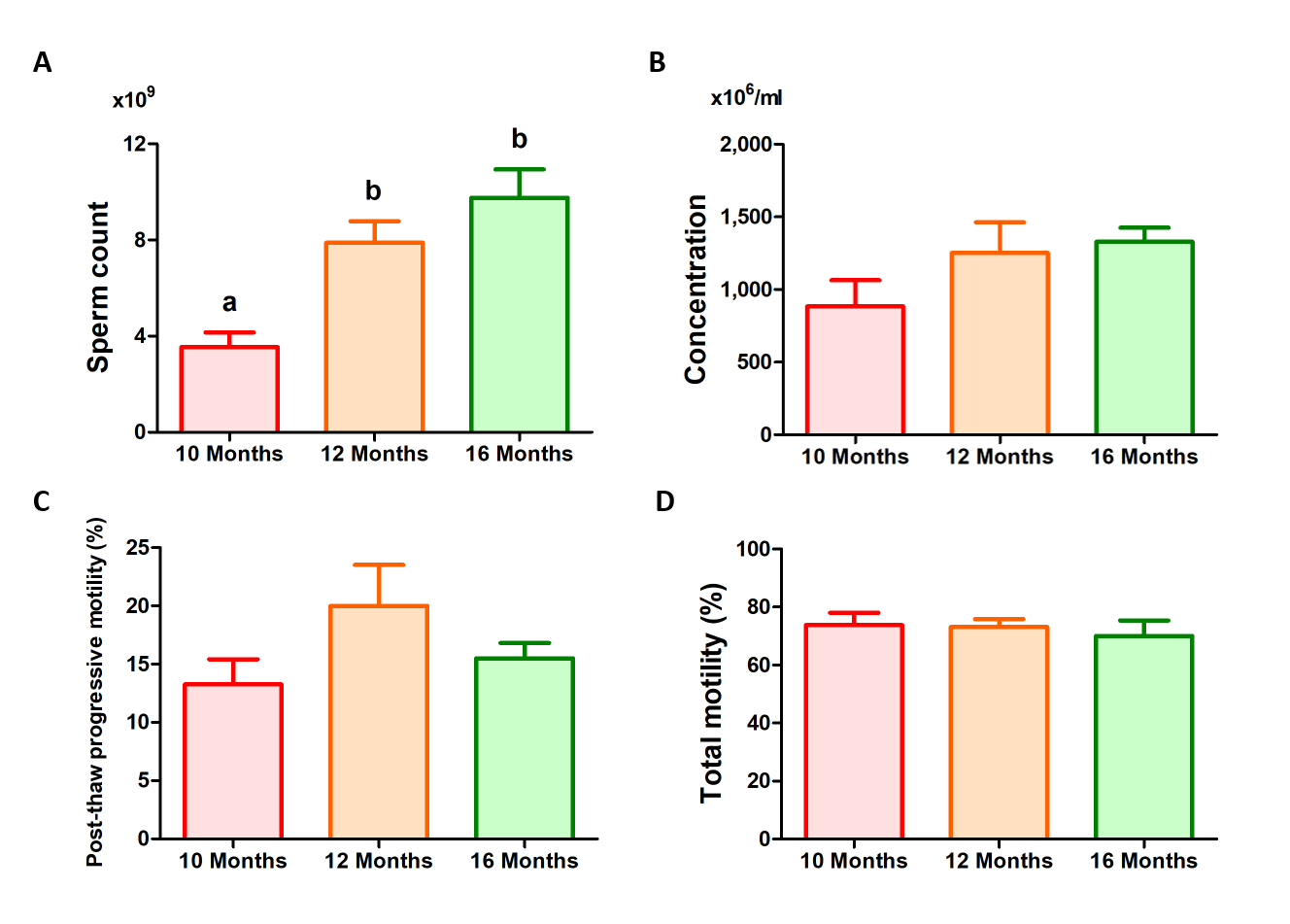

Supplement: Supplementary file 1 — Additional file 1: Figure S1. Semen quality of bulls at age of 10, 12 and 16 months. A: Sperm count; B: Sperm concentration; C: Total motility; D: Post-thaw progressive motility. Bars show the mean ± standard error; different letters represent p-value < 0.05. [file 12864_2020_7206_MOESM1_ESM.tif]

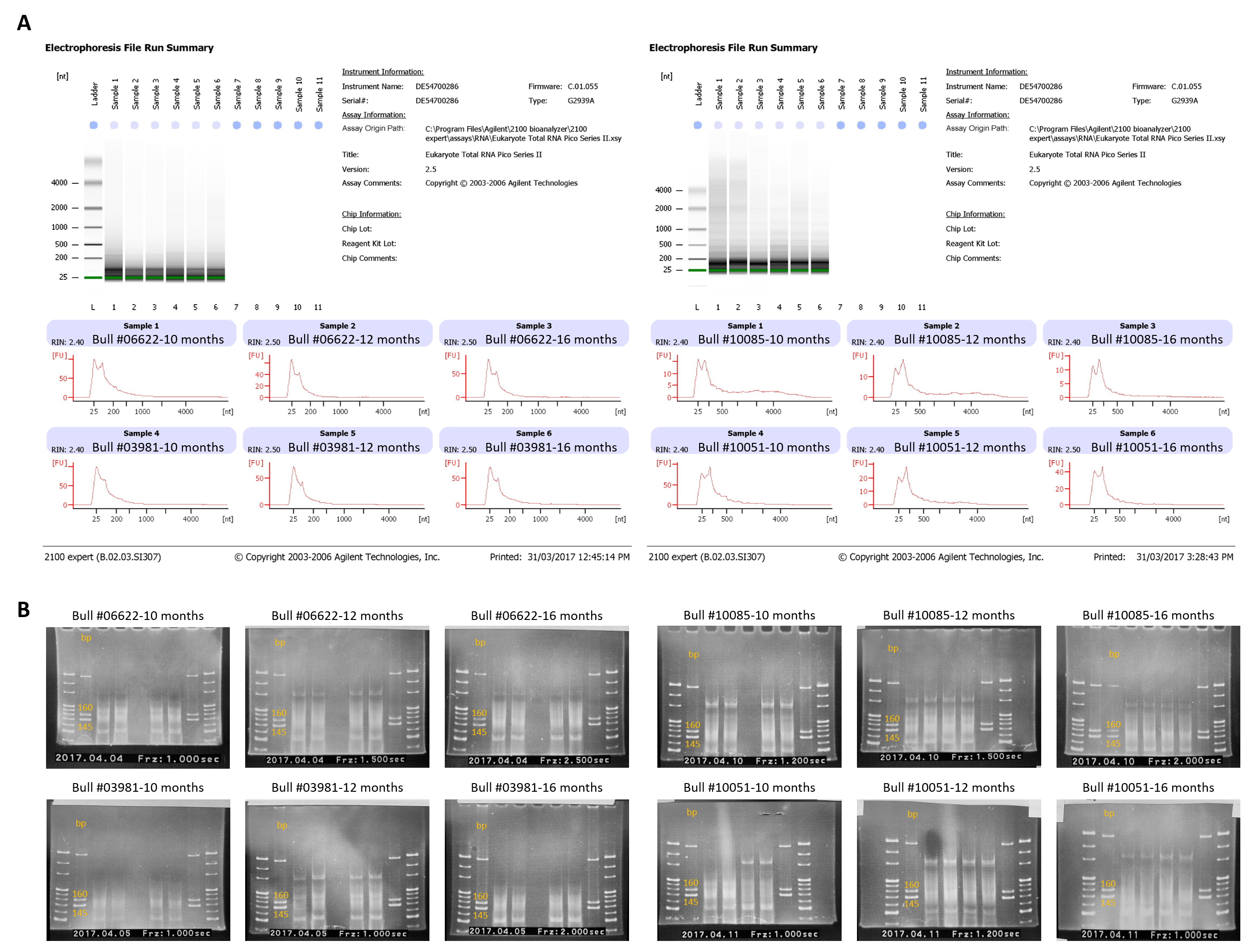

Supplement: Supplementary file 2 — Additional file 2: Figure S2. RNA patterns of sperm from four bulls at 10, 12, and 16-months old. A: Summary of bioanalyzer results of total RNA extracted from sperm. B: Summary of 6% Novex TBE gel results after libraries preparation. Adapters of 125 nt were added, small noncoding RNAs as well as miRNAs were cut and purified for sequencing. [file 12864_2020_7206_MOESM2_ESM.tif]
